# Transmission electron microscopy analysis of UV laser implanted gold nanoparticles and their influence on photoluminescence enhancement from silicon nanocrystals

**DOI:** 10.1186/s11671-025-04263-1

**Published:** 2025-05-16

**Authors:** Lukas Janos Richter, Ulrich Ross, Michael Seibt, Jürgen Ihlemann

**Affiliations:** 1 Institute for Nanophotonics Göttingen e.V., Hans-Adolf-Krebs-Weg 1, 37077 Göttingen, Germany; 2 University of Göttingen, IV. Physical Institute—Solids and Nanostructures, Friedrich-Hund-Platz 1, 37077 Göttingen, Germany

**Keywords:** Si-Nanocrystal, Silicon, Photoluminescence, Plasmonic particle, Gold nanoparticle, Excimer laser, TEM

## Abstract

The photoluminescence of silicon nanocrystals is significantly enhanced via laser-based implantation of gold nanoparticles into silicon suboxide. This study is to explore in detail the nanoscale interactions between silicon nanocrystals embedded in the oxide matrix and gold nanoparticles through transmission electron microscopy. The influence of gold nanoparticle size and distance from the silicon nanocrystals is investigated and correlated with photoluminescence enhancement. A more than threefold enhancement in photoluminescence of silicon nanocrystals is observed, depending on the size and separation distance of the gold nanoparticles, demonstrating the efficiency of this straightforward and rapid laser-based method.

**Supplementary Information:**

The online version contains supplementary material available at 10.1186/s11671-025-04263-1.

## Introduction

Silicon nanocrystals (SiNc) are a prominent candidate to integrate photonic functionality into silicon based microelectronic devices. They offer the potential to emit light with high efficiency, despite the indirect band gap of silicon [1, 2]. This is attributed to several phenomena, including carrier confinement in SiNc at the nanoscale (~ 5 nm) [2–7], defect centers [8–11], or molecular complexes [8, 12], to name a few. These possibilities and the prominence of silicon in semiconductor science and technology resulted in extensive research on silicon nanocrystals. In addition to light emission, other potential applications have been investigated, including biomedical sensors, enhancement of photovoltaic solar cell efficiency, and SiNc-based flash memory devices [1, 13].

The fabrication of SiNc is possible by a number of methods. Briefly, SiNc can be produced by wafer dissolution [14] or generating substoichiometric silica (SiO_x_, x < 2) using silicon ion implantation into quartz [15–17], deposition of silicon-rich layers by vacuum evaporation [18–20], sputtering [21], or plasma-enhanced chemical vapor deposition [22–24], followed by thermal or laser-induced phase separation [25]. The fabrication method influences the resulting size distribution of SiNc, their surrounding matrix as well as defect states and hence the resulting light emission. The latter is usually experimentally studied by photoluminescence (PL) spectroscopy [18, 22] (for further details, see e.g. cited literature).

To enable the transition of SiNc from laboratory-scale research to industrial applications, further improvements in light emission efficiency are required. Although SiNc exhibit efficiencies several orders of magnitude higher than bulk silicon, this remains insufficient for real-world applications [1]. Various approaches have been developed to enhance light emission efficiency. To increase the non-radiative lifetime, attempts are made to eliminate defect states that lead to non-radiative recombination, for example by hydrogen passivation [26]. Surface structuring has also been explored to enhance light outcoupling efficiency in highly refractive SiNc-containing layers. More detailed information about these methods can be found in the literature [27, 28].

One prominent approach to increasing light emission efficiency is the use of plasmonic nanoparticles [15, 29–32]. The spontaneous emission rates of SiNc are influenced not only by their intrinsic properties but also by their surrounding environment. Plasmonic nanoparticles in the vicinity of SiNc change the optical density of states of SiNc and thus result in an increased transition rate, i.e. shorter radiative lifetime [32]. Additionally, energy transfer to surface plasmons at the plasmonic nanoparticle can be radiatively coupled out, reducing non-radiative losses [15].

Previous studies have explored the role of plasmonic nanoparticles in enhancing the emission of SiNc, emphasizing the importance of factors such as the type of metal, nanoparticle size, shape, and their separation distance from the SiNc in determining the enhancement effect. Gold nanoparticles (AuNp) are commonly used due to their stability and resistance to oxidation [15, 29, 32]. Optimal plasmonic resonance and spectral overlap between the AuNp and the SiNc emission wavelength are essential for maximum enhancement [32]. The separation distance between the SiNc and the AuNp is crucial: a distance that is too small can lead to PL quenching, whereas a too large distance diminishes the intensity of the local field [15].

Several studies have investigated these parameters using sophisticated techniques. Biteen et al. demonstrated that a nanoporous gold film, fabricated by dealloying a silver-gold alloy, enhances the PL of the SiNc. SiNc were fabricated by Si^+^ ion implantation into SiO_2_ and subsequent high-temperature annealing. By varying the thickness of the SiO_2_ layer separating the gold and the SiNc, an optimal separation distance for PL enhancement was found to be around 10 nm [15]. Goffard et al. used multilayer SiO_2_-SiO structures and thermal annealing to produce SiNc. PL enhancement by Au nanodisks, fabricated through electron-beam lithography, demonstrates that both the distance between SiNc and Au nanodisks, as well as spectral overlap, influence PL enhancement [32]. Sugimoto et al. reported PL enhancement of colloidal SiNc by colloidal gold nanorods in an aqueous solution [33]. Köthemann et al. analyzed PL enhancement of SiNc by lithographically fabricated gold nanoantennas. SiNc are prepared by high-temperature annealing of alternating layers of SiON and SiO_2_ layers. When gold nanoantennas are placed directly on top of the SiNc-containing layer, no PL enhancement is observed. It is suggested that the Au nanoantennas act as non-resonant scattering centers. Only after overgrowing the Au nanoantennas with an additional SiNc-containing layer PL enhancement is observed [31]. Also, in the case of optical excitation, Gardelis et al. propose an indirect excitation of SiNc by surface plasmons from silver nanoparticles in their vicinity to explain experimentally observed enhanced PL emission [34].

In this report, the method of laser implantation of AuNp into the SiO_x_ matrix is applied to investigate the influence of the distance between SiNc and AuNp on PL enhancement through plasmonic coupling. SiO_x_ layers (x ≈ 1) are produced by thermal evaporation of silicon monoxide on quartz substrates, followed by UV excimer laser irradiation to incorporate AuNp into the SiO_x_ matrix. SiNc are formed within the SiO_x_ layers through high-temperature annealing. By varying the annealing atmosphere, we control parameters such as the AuNp size distribution and separation distance from the SiNc. The resulting SiNc are analyzed using PL spectroscopy and transmission electron microscopy (TEM).

Unlike other more complex and time-consuming techniques, our laser-based method is fast and straightforward. Despite its simplicity, our approach yields similar or even better results in terms of photoluminescence enhancement, demonstrating its potential as an efficient alternative for industrial applications.

## Methods

### Sample preparation: SiNc fabrication

SiNc are produced by thermally induced phase separation of silicon-rich silicon oxide. The required samples are prepared as follows:

Quartz substrates are coated by thermal evaporation of silicon monoxide (SiO) in vacuum (~ 10^–6^ mbar) with an evaporation rate of 1 nm/s. To ensure high adhesion of the coating, substrate surfaces are sputtered for 30 s using argon ions, followed by the coating process, both performed at a substrate temperature of 300 °C. A slight oxidation of the suboxide during the process increases the oxygen content, resulting in SiO_x_ with a composition slightly above the monoxide (1 < x < 2), with a typical value of SiO_1.08±0.02_ as previously reported by us [27]. For this study, a SiO_x_ coating with a thickness of 1 µm was deposited.

Phase separation of the SiO_x_ coating for SiNc formation is induced at 1050 °C in a quartz tube as part of a Nabertherm R50/250/13 furnace with a P330 controller. A gas flow through the quartz tube ensures a controlled atmosphere. During the temperature ramps, nitrogen flow with 99.999% N_2_ reduces oxidation. The high temperature (1050 °C) is kept constant during the annealing time of 3 h. Varying the gas atmosphere during this time allows controlled surface oxidation, i.e. a controlled thickness of an SiO_2_ layer free from SiNc. To achieve oxidation, air is used as the oxidizing ambient. While there is always a slight oxidation of the surface, even in a nitrogen atmosphere, the minimum oxide thickness is obtained by high-temperature annealing for 3 h in nitrogen, while the highest oxide thickness in our experiments was obtained by high-temperature annealing for 3 h in air. Oxide thickness variation between these extremes is possible by splitting the time into periods with different gas flows, e.g. 1 h of air flow and 2 h nitrogen flow.

**Fig. 1:**
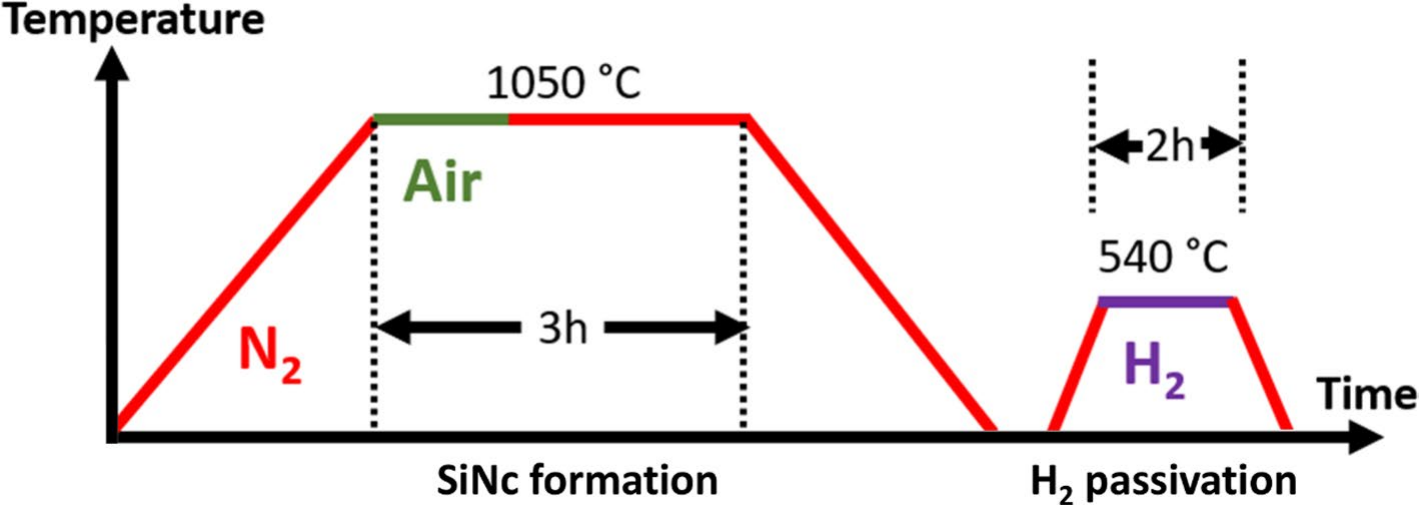
Schematic depiction of the furnace processes. SiNc production is performed at 1050 °C. During the temperature ramps, the furnace tube is purged with nitrogen (shown in red). While the temperature is kept constant for 3 h, the furnace tube is purged either with nitrogen only or temporarily/continuously with air (shown in green). This allows the oxidation of the samples to be controlled. Following the high-temperature process, hydrogen passivation (shown in purple) is carried out at 540 °C for 2 h, whereby the furnace tube is flushed with nitrogen again during the temperature ramps

Subsequent to the high-temperature annealing, hydrogen passivation for two hours at 540 °C was performed to reduce non-radiative recombination at defect states. A forming gas with 5% H_2_ and 95% N_2_ was used during this step. The gas flow rates for all processes were calculated to purge the furnace volume approximately 1.5 times per minute. The entire process is shown schematically in Fig. 1.

### Sample preparation: AuNp implantation by UV laser irradiation

Prior to the high-temperature annealing, AuNp were implanted into the SiO_x_ using UV laser irradiation. This technique has already been described in previous work [29, 35]. For this process, 20 nm of gold was deposited on the SiO_x_ coated samples using a sputtering chamber (Emitech K550). Irradiation by 10 pulses of an ArF-excimer laser (typical pulse length 20 ns; LPX300, Lambda Physik; $\uplambda =193\text{ nm}; \text{f}=10\text{ Hz})$ leads to implantation of the gold into the SiO_x_. A beam homogenizer was employed to generate uniform fluence profiles of 1 × 1 mm^2^, achieved through projection of the homogenized spot by an imaging lens with a 10:1 demagnification. Fluence variation was achieved with a variable attenuator and energy measurements with a pyroelectric sensor (Ophir PE25BF-C). To optimize the AuNp implantation process, the fluence was varied and determined to be optimal at 200 mJ/cm^2^, which was then maintained as a constant value. After UV laser implantation, as described in the previous section, high-temperature annealing is performed for SiNc production. Any remaining gold on the surface is subsequently cleaned by manually wiping the surface with ethanol. The process is also explained in our previous work with an additional process sketch [29].

### Sample analysis

The light emitting properties of SiNc are analyzed by PL measurements. PL excitation is performed by a continuous laser diode (Coherent OBIS 405 LX 100 mW; $\uplambda =405\text{ nm}$) illuminating a spot with 220 µm diameter homogeneously at a power of 0.85 mW. The power of the excitation light is measured with a photo diode (PD300-3 W-V1, Ophir). The PL light from the whole 220-µm-spot is measured in reflection using a lens (LUC PlanFL N 40x/0.6, Olympus). The excitation light is blocked by a dichroic mirror and a longpass filter. Spectral analysis is performed using two customized fiber-coupled grating spectrometers (NIRQuest + 1.7 and HDX, both Ocean Optics). The setup was calibrated using a calibration lamp (HL-2000-LL, Ocean Optics). The measurement data is not smoothed, but the data is multiplied by a numeric factor to ensure a smooth transition of the curves of the two different spectrometers. A sketch for the setup can be found in [29].

Measurements of SiO_x_- and SiO_2_-thickness and transmission data were recorded with an optical layer thickness measurement device (Filmetrics F20-UV with LS-DT2 light source, Filmetrics). Detailed surface analysis was performed using a scanning electron microscope (SEM, Zeiss EVO MA10, Zeiss).

In order to further investigate the samples by transmission electron microscopy (TEM), cross-section lamellas were prepared using standard focused Ga^+^ ion beam preparation (Thermo Fisher Helios DualBeam FIB-SEM). The TEM characterization was performed in a Thermo Fisher Titan 80–300 G2 ETEM operated at 300 kV and equipped with a CEOS CETCOR image corrector as well as an electron energy-loss spectrometer (EELS, Gatan Quantum GIF). In order to enhance the contrast of the SiNc, the instrument was mostly operated in scanning (STEM) mode, and the low-loss spectrum (0–100 eV) recorded through the spectrometer. For all measurements, experimental parameters were: ~0.14 nm probe size (i.e. 10 mrad probe-forming aperture), > 70 mrad spectrometer entrance acceptance angle at 38 mm camera length, 50–80 pA beam current. A typical pixel separation for the STEM-EELS maps was 0.8 nm, as a compromise between spatial resolution and total measurement time. Lastly, the EELS maps were recorded in dual-EELS mode with an energy offset of 13 eV, in order to correct the measurements for slow energy-drift of the spectrometer. The spatial resolution of the resulting STEM-EELS maps is limited by instrumental parameters on one hand, on the other hand the overlap between SiNc along the beam direction as well as the delocalization of the volume plasmons create additional blur between closely-packed particles in the mapping of individual components.

In the low-loss region, SiNc and SiO_2_ exhibit two distinct volume plasmon peaks at approximately 17 and 23 eV, respectively. The AuNp generate a broad feature [36] between 10 and 60 eV; they are clearly visible in the total integrated intensity of spectra due to the related strong high-angle electron scattering. In order to map the presence of SiNc and AuNp inside the SiO_x_ matrix, spectra were decomposed into two Lorentz peaks initially centered at the center of gravity of the SiNc and SiO_2_ features, while a broad Gaussian peak captured both the continuous background as well as the AuNp plasmon signal. In order to improve the quality of the least-square optimization, the parameters were constrained to positive amplitudes and widths, and the shift of the peak centers limited to 1 eV. For a more detailed description of the EELS spectral decomposition, see Figure SM5 in supplementary material (SM). A similar procedure was used and is described in detail in our previous work [27].

## Results

### Oxide thickness by annealing experiments

**Fig. 2:**
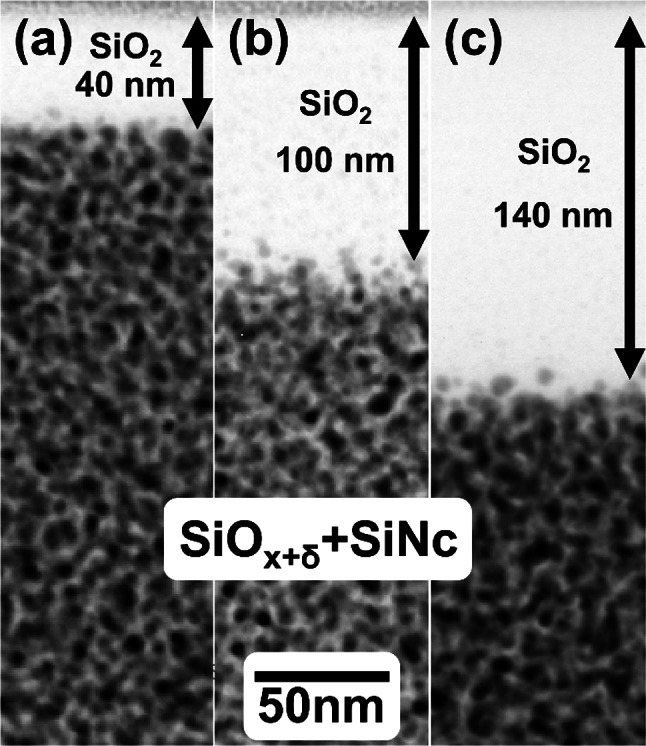
Energy-filtered images extracted from spectrum images (obtained by STEM-EELS) showing reference samples without laser-implanted gold for (**a**) 3 h in N_2_, (**b**) 2 h in air and 1 h in N_2_, (**c**) 3 h in air at 1050 °C. Bright areas are the oxidized surface layers with different thickness indicated besides the arrows. Areas below are SiNc containing SiO_x+δ_

Samples with 1 µm SiO_x_ coatings were used. Different annealing times in air result in differences in oxide thickness at the surface. Prior to the annealing, samples show a native oxide thickness of around 3 ± 2 nm. Post-annealing, oxide thickness was measured using optical techniques and TEM. TEM images (Fig. 2 and SM1 in supplementary material) illustrate oxide layers ranging from 40 to 140 nm, with thicknesses marked by arrows. The region below consists of SiO_x_ decomposed into nanocrystalline silicon (SiNc) and SiO_x+δ_. Although our data do not allow measuring x + δ due to strong overlap with SiNc in electron beam direction, it is reasonable to assume that x + δ≈2 (c.f. [37]). A summary of measurements from both methods is provided in Table 1, demonstrating consistent results between optical techniques and TEM. Additionally, the table includes sample names corresponding to their annealing times in air and nitrogen, e.g., A0_N3 (0 h in air, 3 h in nitrogen).

**Table 1:** Oxide thickness of SiO_x_ samples annealed at 1050 °C for 3 h with a constant gas flow

Sample name	Annealing time in [h]		SiO_2_ oxide thickness measurement [nm]	
	Air	Nitrogen	Optical	TEM
A0_N3	0	3	49	40
A2_N1	2	1	104	100
A3_N0	3	0	140	140

The sample names are chosen according to the annealing time in air and nitrogen. A nitrogen atmosphere is used for the time without air flow and for all annealing ramps

### Photoluminescence measurements

**Fig. 3:**
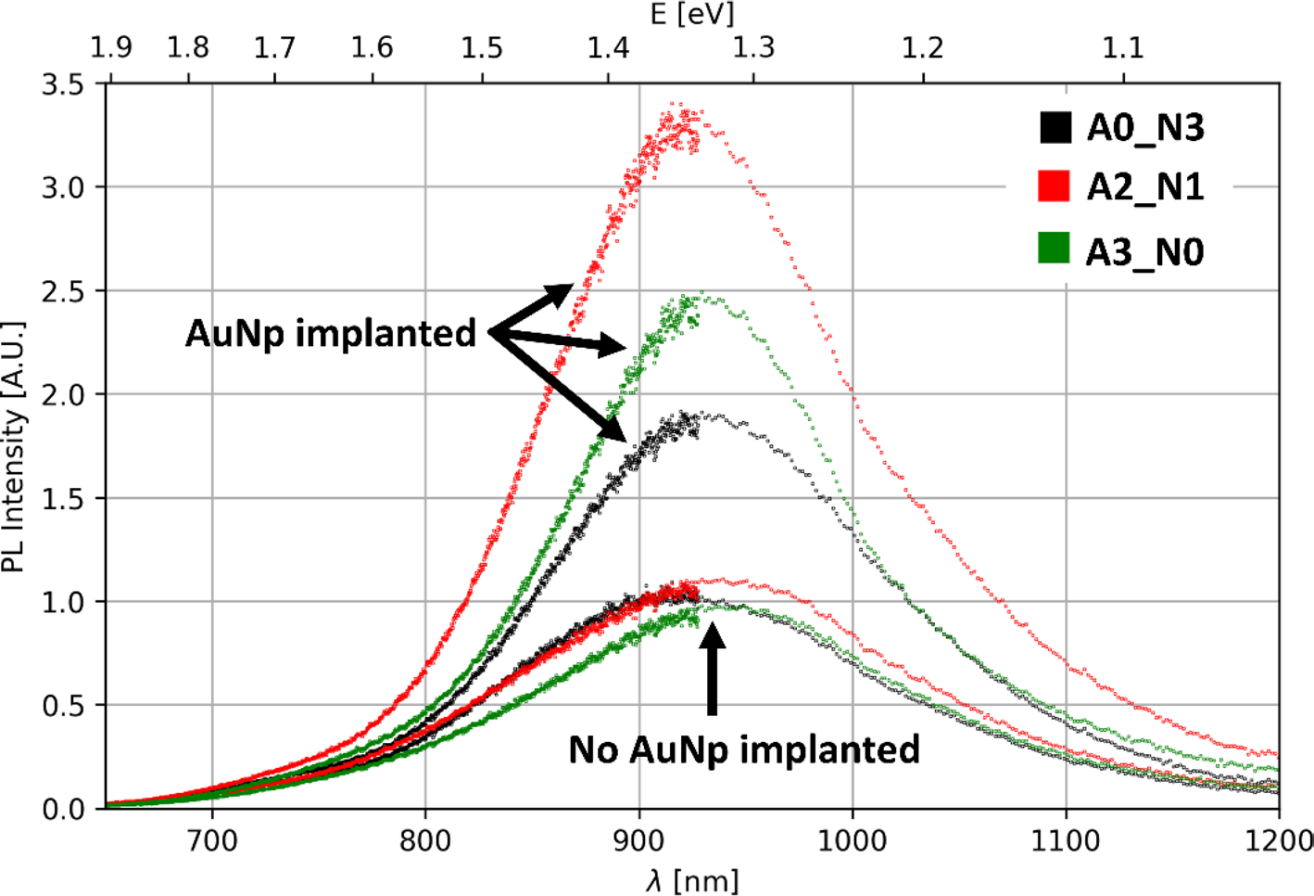
The PL spectra of three samples with 1 µm SiO_x_ coatings on SiO_2_ substrates, which were annealed at 1050 °C for 3 h with individual partial air flow (as indicated on the label), exhibit varying oxide thicknesses on the surface. The three spectra with peak intensities around 1 have no implanted AuNp. The three spectra with higher intensities and the same color show the PL of the same samples with implanted AuNp

Spectral PL measurements show relatively broad light emission between 750 and 1100 nm, peaking around 930 nm. The gas atmosphere during high-temperature annealing causes no major shifts in the spectral position of the PL, but a slight redshift with increasing oxide thickness is observed. In particular, the peak exhibits a shift of 10.7 nm following two hours of air exposure and a shift of 15.1 nm after three hours, in comparison to the sample that was solely annealed in nitrogen. Also, a slight decrease in PL intensity of about 10% is observed for the sample annealed for 3 h in an air environment. This is illustrated in Fig. 3; the spectra are normalized so that sample A0_N3 peaks at an intensity of 1.

All samples with implanted AuNp show significant enhancement of PL. In Fig. 3, they are depicted by the same color as the identically annealed samples without implanted AuNp. An enhancement between 1.8 to 3.3 is measured. Therefore, the intensity of the enhancement greatly depends on the oxide thickness. Sample A0_N3 shows the weakest PL enhancement of 1.8. An annealing for 2 h in air (sample A2_N1) results in a much greater PL enhancement of 3.3. Longer annealing times in air (sample A3_N0) lower the PL enhancement again to a factor of around 2.5.

### Analysis of laser implanted gold nanoparticles

**Fig. 4:**
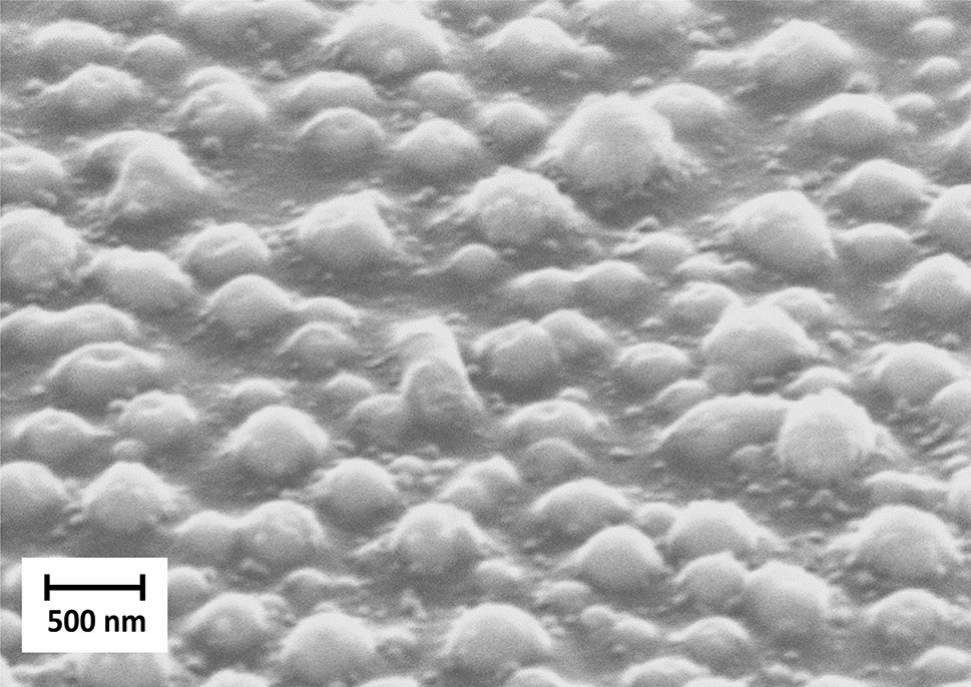
Scanning electron microscopy (SEM) image of a sample surface with a 1 µm SiO_x_ coating on a SiO₂ substrate. Gold nanoparticles (AuNPs) were implanted into the SiO_x_ surface. The hills in the image represent individual AuNp, which are only partially buried. The image was captured at a viewing angle of 60° relative to the surface. The sample was annealed at 1050 °C in nitrogen and cleaned using ethanol wiping. The AuNp were implanted with 10 laser pulses at a wavelength of 193 nm and a fluence of 200 mJ/cm^2^

Typical absorption spectra of annealed samples with implanted AuNp can be found in a previous work of ours [29, 35]. They exhibit an absorption peak at about 550 nm. Individual AuNp buried in the silicon oxide can be seen in the SEM image in Fig. 4. The surface of a SiO_x_ sample with implanted AuNp is shown at a viewing angle of 60°. 10 laser pulses at a fluence of 200 mJ/cm^2^ were applied. The sample was high-temperature annealed and subsequently cleaned. Large AuNp are apparently only partially buried. Their size ranges from roughly 50 nm to 500 nm.

### Microstructural analysis using TEM

**Fig. 5:**
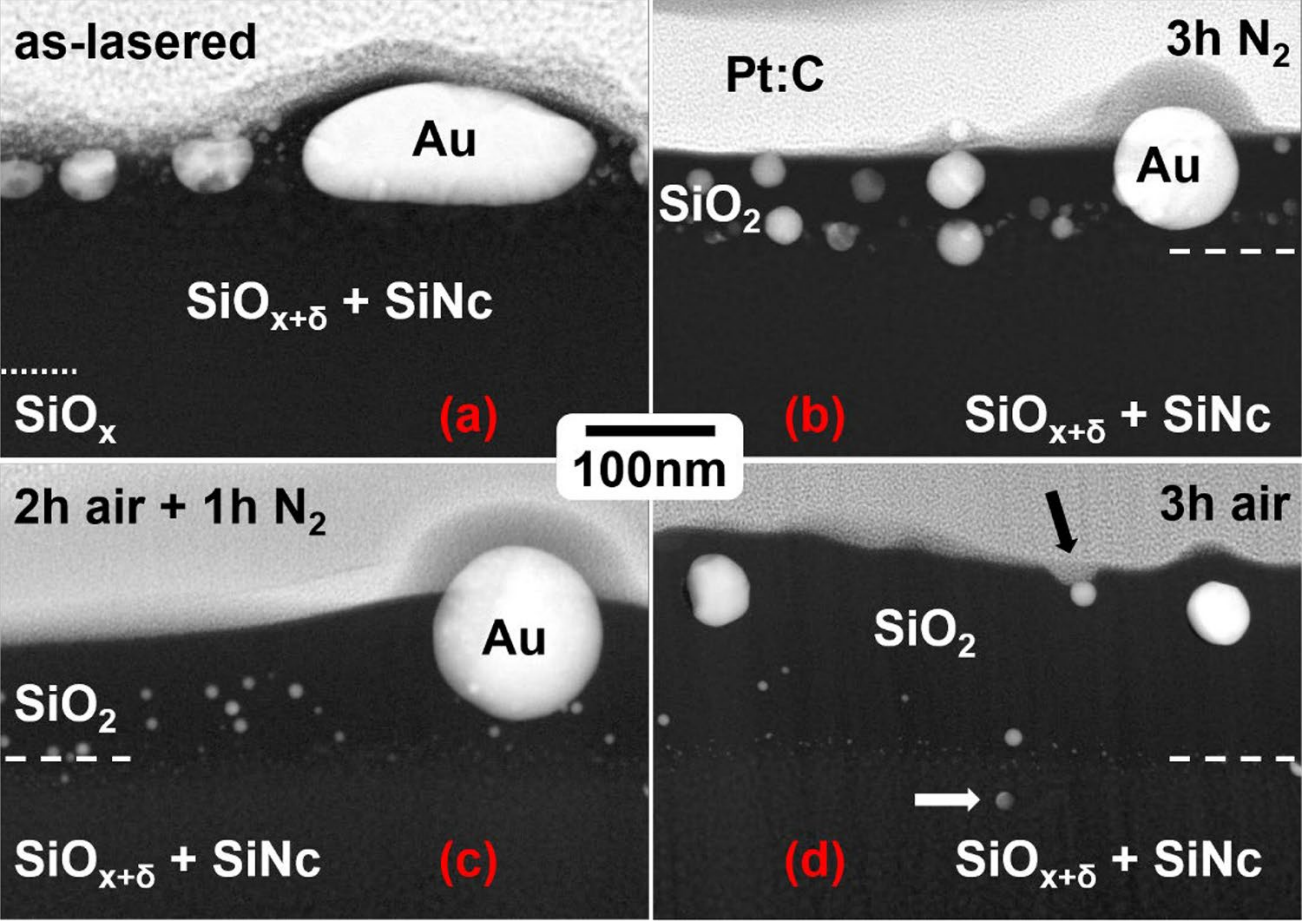
STEM-ADF images of Au laser-implanted samples after additional annealing. **a** As-lasered sample: Au nanoparticles (AuNp) are located beneath the surface, followed by a partially decomposed SiO_x_ layer (denoted as ‘SiO_x+δ_ + SiNc’) and unreacted SiO_x_ below the dotted line. Coverage by SiO_2_ is observed around larger Au particles. **b** After 3 h of annealing in N_2_ (A0_N3): slight oxidation occurs in the region above the dashed line, with AuNp remaining beneath the surface and near the interface between SiO_2_ and decomposed SiO_x_. **c** After 2 h in air and 1 h in N_2_ (A2_N1): an increased SiO_2_ thickness is evident, accompanied by smaller AuNp at the interface and within the SiO_2_ layer. **d** After 3 h of annealing in air (A3_N0): different oxide thicknesses (white dashed lines) and distinct depth distributions of AuNp are observed. Pt:C denotes the coating necessary for TEM lamella preparation. Occasionally, AuNp associated with an open pore (black arrow) are observed as well as AuNp located within the (partially) decomposed SiO_x_ (white arrow). Laser parameters for AuNp implantation: 10 pulses at 200 mJ/cm^2^

Prior to a detailed description and discussion of the different processing conditions on AuNp and SiNc size and distribution, Fig. 5 presents an overview of STEM annular darkfield (ADF) images for direct visual comparison. Bright particles correspond to AuNp, which tend to have a spherical shape (Fig. 5b–d) with the exception of the as-lasered sample. In addition, besides AuNp directly beneath the surface (marked here by the interface to the Pt:C protection layer for FIB preparation), AuNp move into the growing SiO_2_ layer. Furthermore, STEM images indicate a continuous loss of Au mainly substantiated by a reduced density of larger particles (see also below).

#### Laser implanted AuNp: no annealing

The initial stage of our processing scheme is represented by laser implanted AuNp without subsequent annealing. A STEM-ADF image of such a sample is shown in Fig. 5a. The bright particles represent AuNp close to the surface. It can be seen that the AuNp are generally not spherical, with heterogeneous sizes of around 10 nm up to a few hundred nanometers. The AuNps were implanted in the uppermost region of the coating. A substantial portion of the implants were only partially embedded, resulting in incomplete coverage by silicon oxide (cf. Figure 4).

**Fig. 6:**
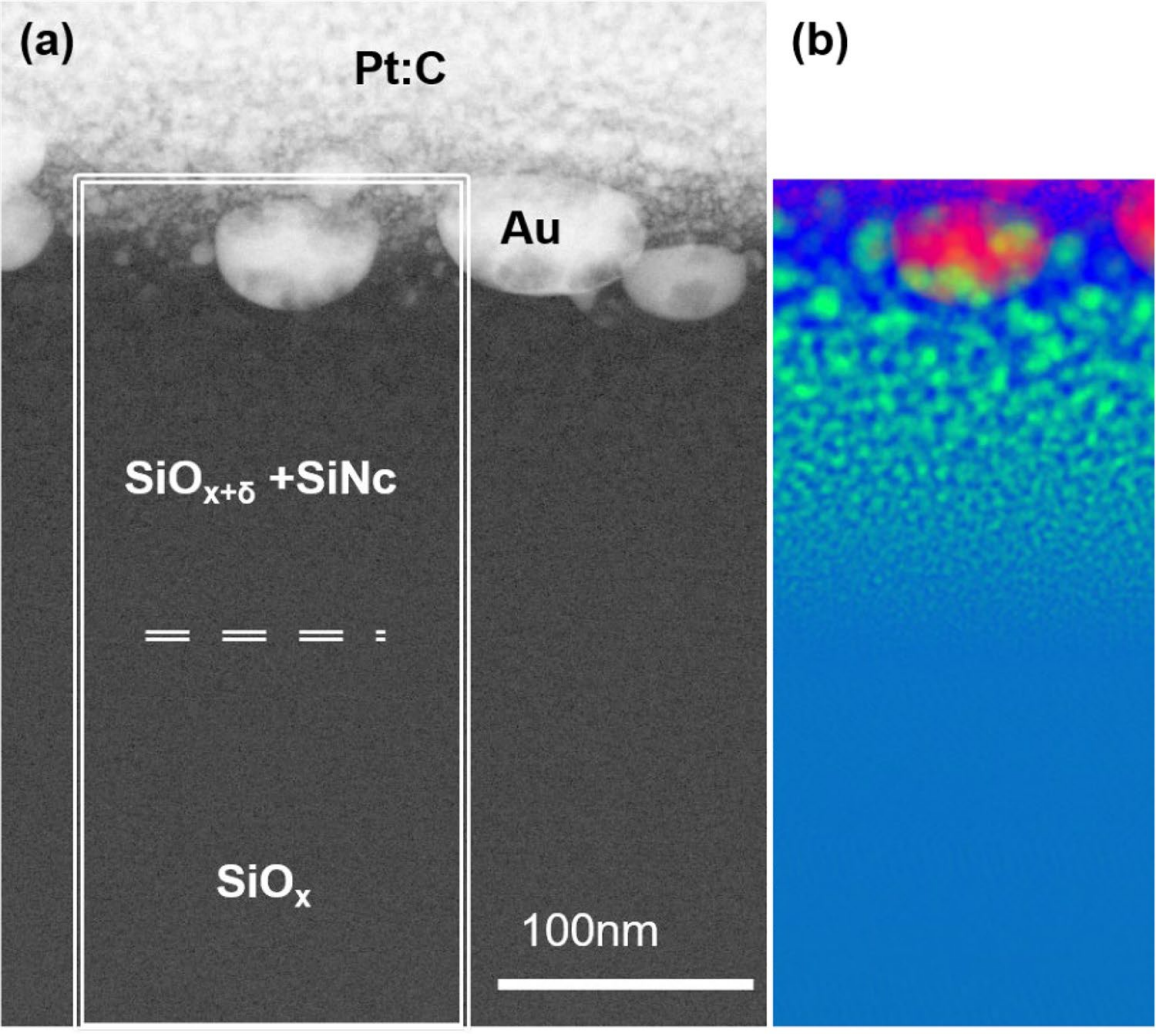
Microstructure of as-lasered samples. **a** STEM-ADF showing AuNp buried beneath the surface (example labeled ‘Au’). **b** Color-coded contributions of Au (red), Si (green), SiO_2_ (dark blue), and unreacted SiO_x_ (light blue) to the EELS signal obtained by fitting a spectrum image. The dashed line in **a** approximately marks the transition of (partly) decomposed SiO_x_ to unreacted SiO_x_. Laser parameters for AuNp implantation: 10 pulses at 200 mJ/cm^2^

Results of EELS deconvolution by plasmon fits are shown in Fig. 6, where (a) indicates the region subjected to spectrum imaging. The resulting color-coded image (Fig. 6b) provides evidence that nanocrystalline silicon (SiNc) was generated through laser irradiation, as evidenced by the green spots shown in part (b) of Fig. 6. A strong size gradient from large to small particles can be seen with increasing distance from the surface. Eventually the particles become smaller than the pixel resolution, and there is a transition to the SiO_x_ layer without visible crystalline silicon. Within the suboxide, smaller (< 1 nm) Si-rich clusters may still be present. These deeper regions do not appear to be modified by the laser irradiation.

#### Annealing of laser implanted AuNp: 3 h in N_2_ (A0_N3)

During 3 h annealing at 1050 °C in a nitrogen atmosphere, a SiO_2_ thickness of around 45 nm was formed due to residual oxygen. The oxide thickness of the annealed samples with laser implantation is systematically slightly larger than the reference measurements, which can be understood to occur due to the laser-modification of the SiO_x_ material resulting in coarse SiNc at the very top of the modified layer. TEM analysis was performed in parts of the samples without AuNp implantation (called reference, TEM images not shown) and in parts with implanted AuNp. The reference sample shows a homogenous density and size distribution of SiNc over the whole depth of the SiO_x_ layer except slight size gradients close to the layer/substrate interface. No SiNc can be found in the top SiO_2_ layer resulting from oxidation.

**Fig. 7:**
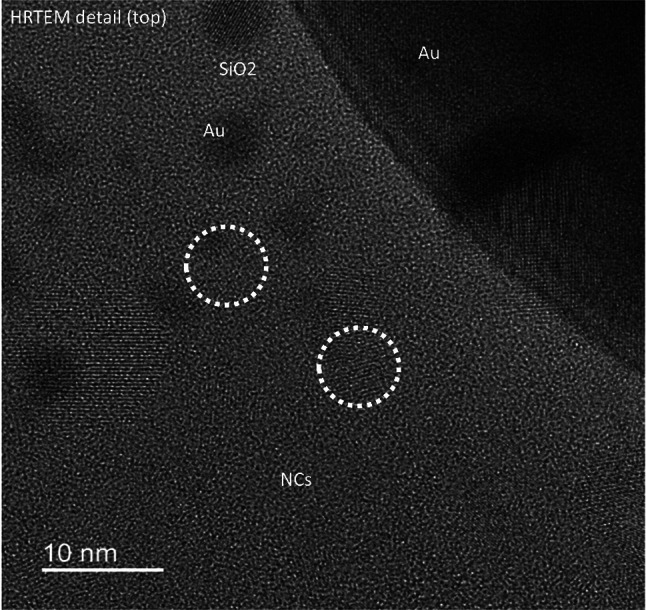
HRTEM of a SiO_x+δ_ sample annealed for 1 h in nitrogen atmosphere at 1050 °C with implanted AuNp. Several SiNc can be identified by the periodic lattice, two SiNc are marked by white dashed lines. Laser parameters for AuNp implantation: 10 pulses at 200 mJ/cm^2^

In the sample with laser implanted AuNp, the oxidized top layer with 45nm thickness is free from SiNc. Large variants up to 13 nm in diameter of the latter were formed close to the interface between SiO_2_ and the decomposed SiO_x_ (labeled SiO_x+δ_ + SiNc) (see SM, Figure SM2). The size of the SiNc gradually decreases away from the interface down to a depth of 140 nm resulting in a second interface to the “bulk” SiNc. Below 140 nm from the surface, the SiNc show the same particle size as in the reference sample. This “bulk” size distribution has been determined to be 3.8 to 11.5 nm [27]. The lower bound of size for clearly identifiable SiNc in the laser-affected region is 3 nm, although the gradient towards increasing SiNc size towards the top is continuous. The largest connected SiNc clusters at the interface to the top SiO_2_ in the sample without annealing can measure up to 18 nm (Fig. 6). Regarding the AuNp: Large AuNp (around 100 nm) may occasionally only be partially buried. These large particles can also be seen by surface analysis, for example in the SEM image in Fig. 4. Besides these large particles, medium sized particles in the range of 20 nm to 100 nm are present. A high magnification STEM-ADF image (Fig. 5b) shows AuNp to be distributed within the SiO_2_ layer down to the interface SiO_2_—SiO_x_ with a relatively uniform distribution. No particles have significantly passed the interface into the silicon-rich bulk. Most particles show diameters below 40 nm, with the majority being very small (below 10 nm). It can be observed that particles of a smaller size are situated in close proximity to the interface between SiO_2_ and SiO_x_. The rest of the AuNp have distances between 20 and 40 nm to the SiO_x_ layer. For better clarity, the distance and size measurements are also summarized in Table 2. This data is provided as an estimate and is intended solely for general reference. Overall, at the smallest SiO_2_ layer thickness, the distance between the AuNp and the SiNc is very short or the AuNp are even in contact to the SiNc. This can also be seen in the HRTEM image in Fig. 7. SiNc can be identified by the periodic lattice in SiO_x_, two individual SiNc are marked with white dashed lines as examples. AuNp are very close, or even in contact, to the SiNc. In some instances, the SiNc and AuNp are observed to overlap, with both materials projected in front of each other within the TEM lamella.

**Table 2:** Manual count of AuNp per size class derived from TEM analysis of the samples

Sample name	Lamella width [µm]	AuNp count per size class, normalized to lamella width [1/µm]													AuNp—SiNc distance [nm]
		< 10 nm	10 nm	20 nm	30 nm	40 nm	50 nm	60 nm	70 nm	80 nm	90 nm	100 nm	110 nm	120 nm	
A0_N3	3.58	44	1.7	10.3	6.7	0.28	0	0.28	0.84	0.84	0.56	0.28	0	0.28	20 to 40
A2_N1	5.13	43	0	0	0.58	0.2	0.2	0.2	0.39	0.39	0.58	0	0.58	0	30 to 50
A3_N0	0.9	118	7.8	3.3	1.1	0	2.2	0	0	0	0	0	0	0	100

Counts are normalized to the lamella width. The distance between AuNp larger than 10 nm and SiNc containing SiO_x_ layer is shown. Similar TEM images as in Figure SM1 (in supplementary material) were used to provide this data (images not shown). Please note that this data is intended as an approximation and should be used for general orientation only

#### Annealing of laser implanted AuNp: 1 h in N_2_ followed by 2 h in air (A2_N1)

Annealing in air for two hours and in N_2_ for one hour increases the oxide thickness to about 100 nm (cf. Figure 2 and SM1 in supplementary material). The reference sample, without implanted AuNp, again shows homogenously distributed SiNc with minor size gradients at the interfaces (TEM images not shown).

The sample with laser implanted AuNp shows a wide range of AuNp sizes (cf. Figure 5c). A few half-buried particles with roughly 100 nm size are close to the surface. Medium sized AuNp are preferentially distributed along the lower half of the SiO_2_ surface layer (cf. Figure SM1 in supplementary material). Close to the interface SiO_2_—SiO_x+δ_ many small AuNp (< 10nm) are present. The fraction of these small particles seemed to have increased compared to the sample with shorter annealing time in air (cf. Figure 5b). Additionally, the observation that larger AuNp partially dissolve into numerous very small AuNp (see Figure SM3 (a) in supplementary material) lends further support to this hypothesis. The distance of the medium-sized AuNp (with a diameter of between 30 and 110 nm) from the SiO_x_ interface is approximately 30–50 nm. Again, no significant amount of AuNp can be found below this interface. For ease of reference, the key distance and size measurements are summarized in Table 2.

#### Annealing of laser implanted AuNp: 3 h in air (A3_N0)

The largest SiO₂ layer was formed by high-temperature annealing for 3 h in air only, resulting in a thickness of 140 nm. Again, homogenously distributed SiNc are present in this sample with minor size gradients at the interfaces (TEM images not shown). The TEM image of the sample with laser implanted AuNp is shown in Fig. 5d. The trend of decreasing AuNp size persists. Many small particles (< 10 nm) can be found at the interface SiO_2_—SiO_x+δ_ (cf. Figure SM1 in supplementary material). Some AuNp with sizes around 10 nm can be found in the lower half of the SiO_2_ layer. Only a few larger AuNp (20–50 nm) can be found close to the surface with distances around 100 nm to the SiO_x+δ_ interface. To facilitate comparison, the measured distances and sizes are presented in Table 2. Some AuNp sink towards the SiO_x_ and form pores, as highlighted by the black arrow in Fig. 5d.

To summarize the most important processes with regard to the different annealing times in air: It can be seen that with longer annealing times in air, and thus thicker SiO_2_ layers, a greater proportion of smaller AuNp (< 10 nm) form. These are deposited primarily at the SiO_2_—SiO_x+δ_ interface. Larger AuNp remain close to the surface in all cases. Medium-sized AuNp (20–100 nm) are distributed in the SiO_2_ layer at the shorter oxidation times, but are hardly present in the thickest SiO_2_ layer. The distance of these AuNp to the SiO_x_ interface increases with the oxide thickness. There appears to be a dissolution of the larger AuNp into smaller AuNp.

## Discussion

### PL of SiNc

Firstly, the spectral position and spectral bandwidth of the PL spectra of the SiNc are in good agreement with the literature. Similar spectra have been observed for SiNc with these size distributions [19, 22]. A more detailed analysis of the influence of temperature during high-temperature annealing and hydrogen passivation on the samples, conducted by PL and Raman, can also be found in our previous work [27, 29].

The PL spectra of SiNc samples without implanted AuNp, annealed for different durations, show only minor variations. A slight redshift was observed in samples annealed for longer times in air, i.e. having larger SiO_2_ thickness. After three hours of annealing in air, the PL intensity decreases by approximately 10%. This reduction can be partially attributed to a corresponding reduction in the number of SiNc, as approximately 10% of the sample volume containing SiNc oxidized during the annealing process.

Before examining the enhancement of PL by implanted AuNp, it is necessary to make some observations regarding the SiNc in the samples in which AuNp were implanted by laser: It was observed that an area of SiNc near the surface has a different size distribution than the reference samples. In the sample that was only heated in nitrogen, this region is 140 nm deep (cf. Figure SM2 in supplementary material). This is analogous to the depth of the unheated sample in which AuNp was implanted, wherein crystalline silicon is detectable (cf. Figure 6). This depth also corresponds to the known penetration depth of nanosecond laser pulses used in these experiments, where below a certain depth, sufficient energy deposition no longer occurs, as documented in the literature [27, 38]. It can therefore be stated that laser irradiation can produce crystalline silicon in the range of the significant penetration depth of the laser, whereby the crystalline silicon exhibits a strong size gradient.

The heating process produces different distributions of SiNc in these laser-modified areas compared to samples that have not been modified by a laser. However, the samples that were heated in air for 3 h, no longer show this area, as the oxidization to SiO_2_ consumes the previously formed SiNc (cf. Figure SM4 in supplementary material). The SiNc formed by thermal processes in SiO_x_ regions previously modified by UV laser irradiation show a slight enhancement in PL compared to SiO_x_ regions without UV laser irradiation prior to the thermal process. This can be attributed to an enhanced nucleation rate of the SiNc in the laser affected regions resulting in smaller SiNc (further details can be found in [27]). In this previous study, the effect of additional laser-irradiation on the PL enhancement from SiNc, created by annealing, only accounted for 10–20% at a laser fluence of 500 mJ/cm^2^. Therefore, at the fluence of 200 mJ/cm^2^ used for the Au implantation in this work, this enhancement effect should be even smaller and thus of minor interest for the PL enhancements due to AuNp implantation. Still, this small effect is present when the top 140 nm are oxidized compared to samples with less oxide thickness. This effect might also partially explain the slightly reduced PL of the samples annealed for 3 h in air. In conclusion, the PL enhancement resulting from laser irradiation is relatively minor and considerably less significant than that resulting from AuNp.

### Influence of AuNp to the PL

Implantation of AuNp significantly increases the PL of SiNc in all experiments with a pronounced dependence on the annealing time in air. It was shown by extensive STEM analyses that the oxide thickness on the surface, the AuNp size and distribution as well as the distance between the AuNp and the SiO_x_ interface change with the annealing time in air. A comparison of the distribution, size, and shape of the AuNp in the unheated sample (see Fig. 5 (a) and SM1 in supplementary material) with those of the samples annealed at 1050 °C indicates that the annealing process results in significant material transport. In all cases, the AuNp initially move towards the decomposed SiO_x_ as the oxide layer grows. Additionally, the shape of the AuNp undergoes a transformation, becoming increasingly spherical. It can also be observed that the AuNp diminishes in size, particularly with the longest heating time in air, where the majority of particles are less than 10 nm in size. These small particles appear to migrate with the oxidation front, as they are predominantly detected at the interface with the SiO_x_. The phenomenon of the occasionally observed sinking of AuNp with pore formation (black arrow in Fig. 5d) has been previously described in the literature [39] and linked to equilibrating forces arising from surface tensions of associated interfaces Au/SiO_2_, Au/vacuum and SiO_2_/vacuum. We note here, that this mechanism is not active once AuNp are buried inside SiO_2_ as is essentially the case for all AuNp in our experiments. A further aspect reported in [39] is a continuous loss of Au during annealing, which was attributed to Au evaporation by the authors. In our experiments, Au losses increase with oxidation time while all samples have identical thermal budget indicating evaporation not being the dominant mechanism. Movement of AuNp with SiO_x_ oxidation might be related to Si diffusion through AuNp. In fact, thin Au layers on Si substrates provide diffusion paths for SiO_2_ formation on top of them [40]. Clearly, fully understanding the underlying mechanisms needs to incorporate simultaneously operating processes of SiO_x_ oxidation and decomposition as well as formation of Au:Si alloys, which—considering the Au:Si phase diagram [41]—are most likely liquid at processing temperature.

When it comes to PL enhancement by AuNp, two main factors play a crucial role: the size of the AuNp and the distance between the AuNp and the SiNc. Scattering and absorption simulations based on Mie theory illustrate the size dependence of AuNp behavior [42–46]. Scattering becomes significant only when the particle size is comparable to the wavelength of light. Simulations show that small AuNp (< 10 nm) concentrate scattered electric fields within an extremely localized area near the particle, creating a strong near-field effect. However, for these small AuNp to enhance the PL of SiNc effectively, the SiNc must be located within this narrow high-field region. At such close distances, quenching effects dominate due to nonradiative energy transfer, which reduces PL instead of enhancing it [15, 32]. Furthermore, the absorption efficiency of small AuNp (< 10 nm) is minimal, with optimal absorption occurring at particle sizes of around 50–100 nm. Therefore, small AuNp (about < 10 nm) can be neglected for the PL enhancement.

Our PL measurements can be explained by both the size of the AuNp and the separation distance. For samples annealed in air for 2 h or less, the AuNp exhibit a distribution of larger to smaller particles, which supports PL enhancement given a suitable separation distance. However, for the sample annealed in air for 3 h, most of the AuNp are very small, leading to minimal PL enhancement due to the reasons mentioned above, resulting in lower overall PL enhancement.

In addition to these explanations, the distance of the AuNp to the SiNc also matters. The distance between AuNp and SiNc depends on the oxide thickness, as the AuNp primarily remain in the oxide layer. As the previous paragraph suggests, it makes sense to distinguish the small AuNp (< 10 nm) from the larger AuNp. All samples show very small AuNp primarily at the interface SiO_2_—SiO_x+δ_. As these AuNp are too small for efficient absorption, they can be ignored for PL enhancement. The sample that was annealed in nitrogen only shows an oxide thickness of about 40 nm with separation distances between the AuNp and the SiO_x_ interface of 20–40 nm. Particles with diameters exceeding the oxide thickness may even be in contact to the SiNc and therefore quench the PL by charge transfer [15, 32, 47]. Given their proximity to the SiO_2_—SiO_x+δ_ interface, a considerable number of medium-sized AuNps are situated in close proximity to, or even in direct contact with, the SiNc. This proximity may potentially lead to PL quenching as mentioned above. This may provide an explanation for the smaller enhancement in PL observed for this sample, whereby the sample A2_N1 exhibited a larger PL enhancement. For this sample an oxide thickness of roughly 100 nm was measured. While larger AuNp are still located close to the sample surface, medium sized AuNp (between 10 and 100 nm diameter) are more evenly distributed across the SiO_2_ layer leading to separation distances between 30—50 nm. A greater separation distance between the AuNp and SiNc is evident, with nearly no AuNp being in direct contact with SiNc. The longest annealing time in air (3 h) resulted in the largest oxide thickness of 140 nm. This sample exhibited a reduction in PL enhancement compared to the previous sample. The distance between AuNp and SiNc is approximately 100 nm. As documented in the literature, excessive separation distances have been shown to diminish the PL enhancement [15, 32].

## Conclusion

In summary, laser implantation of AuNp presents an efficient and straightforward method to significantly enhance the photoluminescence of SiNc. The AuNp are incorporated into the SiO_x_ layer in a durable and scratch-resistant manner, providing reliable PL enhancement of SiNc. The enhancement is influenced by several key factors, most notably the separation distance between the SiNc and AuNp. A distance that is too close leads to quenching, while a distance that is too far weakens the local field intensity and reduces the enhancement. Additionally, the size of the AuNp plays a crucial role, as very small nanoparticles have a minimal impact on PL enhancement. As the density of PL-active AuNp decreases significantly during prolonged oxygen supply, fewer AuNp are contributing to the enhancement. This, combined with the larger separation distances, explains the reduced PL enhancement observed in the sample with thick oxygen layer.

In the described experiments, only a small fraction of the nanocrystals in the entire layer are affected by the AuNp. According to Mie theory, the field enhancement near a gold particle of 100 nm diameter decreases to 1/e^2^ in a distance of about 50 nm (in the oxide matrix environment). Thus, only about 5% of the entire layer contribute to the PL enhancement. Based on this, more complex multilayer architectures are conceivable, where the enhancement could be even significantly higher. While the exact mechanisms behind every detail of the enhancement are complex, this study demonstrates that our simple and non-complex laser-based approach yields significant PL enhancement, comparable to more sophisticated methods. This makes our technique a viable option for practical applications where scalability and efficiency are critical.

## Supplementary Information

Supplementary Material 1

## Data Availability

The datasets generated and/or analyzed during the current study are available from the corresponding author upon reasonable request.
