# Supplementary material for: Transmission electron microscopy analysis of UV laser implanted gold nanoparticles and their influence on photoluminescence enhancement from silicon nanocrystals
Source: Discov Nano. 2025 May 16;20(1):82. doi: 10.1186/s11671-025-04263-1 (PMC12084480; doi:10.1186/s11671-025-04263-1)
Supplement: Supplementary file 1 — Supplementary Material 1 [file 11671_2025_4263_MOESM1_ESM.docx]

**Transmission electron microscopy analysis of UV laser implanted gold nanoparticles and their influence on photoluminescence enhancement from silicon nanocrystals**

**L. J. Richter^1^, U. Ross^2^, M. Seibt^2^, J. Ihlemann^1*^**

1 Institute for Nanophotonics Göttingen e.V.; Hans-Adolf-Krebs-Weg 1, 37077 Göttingen, Germany

2 University of Göttingen, IV. Physical Institute – Solids and Nanostructures; Friedrich-Hund-Platz 1, 37077 Göttingen, Germany

* Correspondence: juergen.ihlemann@ifnano.de

**Supplementary Material**

**Low-magnification overview**

Figure SM1 provides STEM-ADF images showing the whole substoichiometric silicon oxide layer (interface to SiO_2_ marked by dashed lines) of samples after laser implantation of Au (a), additional annealing at 1050°C for 3 h in N_2_ (b), 2 h in air plus 1 h in N_2_ (c), and 3 h in air (d). In (a), the dotted line indicates the depth affected by laser irradiation, in (b)-(d) the dotted line marks the interface between oxidized SiO_x_ (SiO_2_) and decomposed SiO_x_.

**
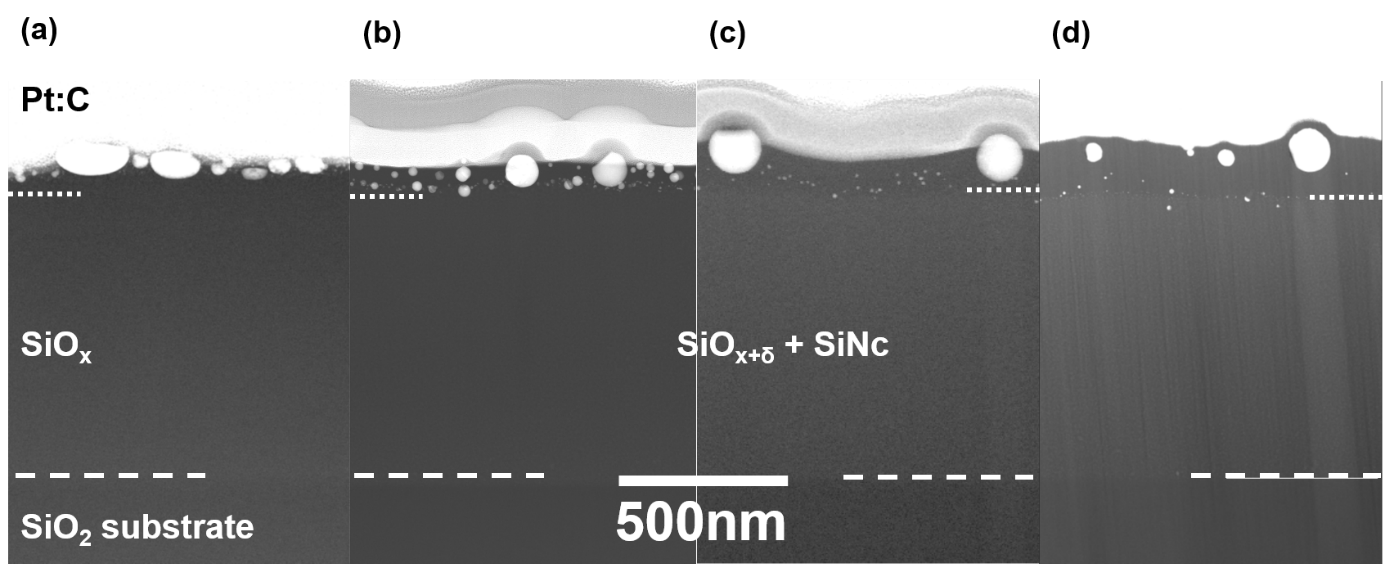
**

Figure SM1: STEM-ADF images showing Au laser-implanted samples after additional annealing: (a) as-lasered, (b) A0_N3, (c) A2_N1, (d)A3_N0. Dashed lines mark the interface between the layer and the SiO_2_ substrate (used to vertically align images). The dotted line in (a) indicates the interface between bulk SiO_x_ and the top layer affected by UV laser treatment. Dotted lines in (b)-(d) mark the position between oxidized (above) and non-oxidized, (partly) decomposed SiO_x_ (below and marked ‘SiO_x+δ_ + SiNc’). Different oxide thicknesses are clearly identified (white dotted lines) as well as different depth distributions of AuNp. The latter are characterized by AuNp directly beneath the surface. Furthermore, a gradual loss of Au is indicated by the decreasing number of large AuNp. The volume expansion of the layer due to oxidation can be clearly noticed from the total layer thickness in (b)-(d), while the decrease in the size of the (laser-modified) SiNc regions is more readily visible in figures SM2-4.

**EELS maps of annealed samples**

Figures SM2-SM4 show EELS maps to provide information about the spatial distribution of Au (red), SiO_2_ (blue) and Si-rich (green) obtained by deconvolution of spectrum images by the fitting procedure described in the main text.


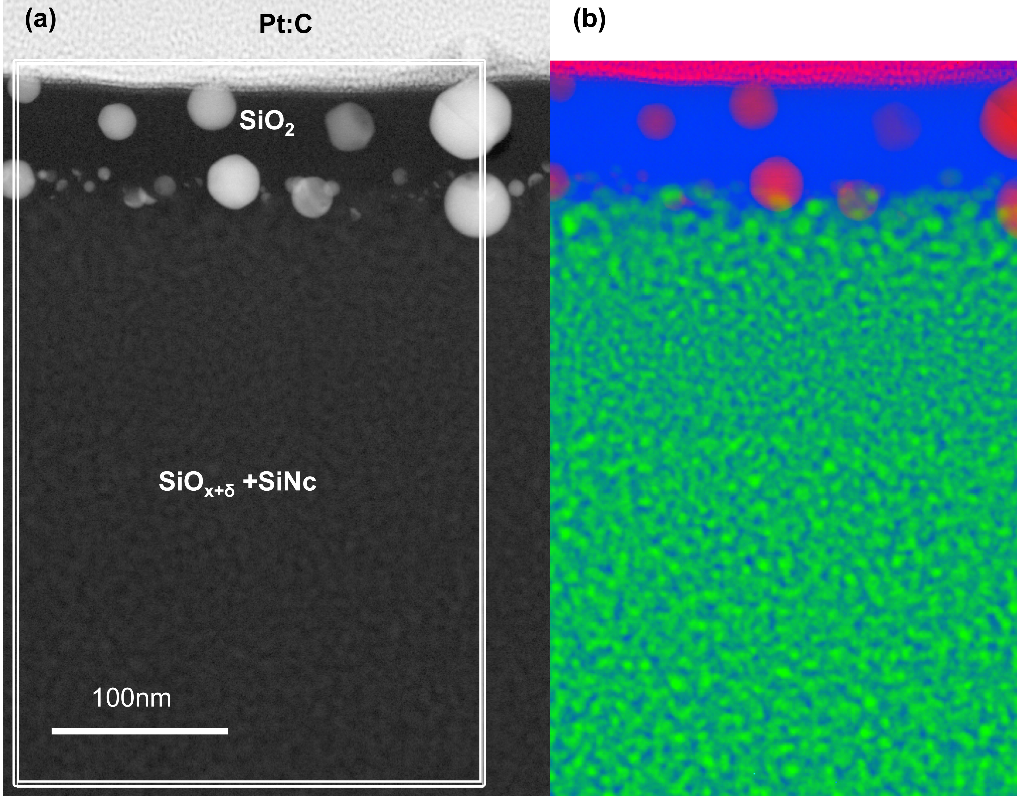


Figure SM2: AuNp distribution after annealing for 3 h in N_2_ (A0_N3). (a) STEM-ADF showing AuNp of different size beneath the surface and at the interface between the oxidized SiO_x_ (labeled as ‘SiO_2_’) and the SiO_x_ decomposed into SiO_x+δ_ and SiNc. (b) Color-coded contributions of Au (red), Si (green) and SiO_2_ (blue) to the EELS signal obtained by fitting a spectrum image.


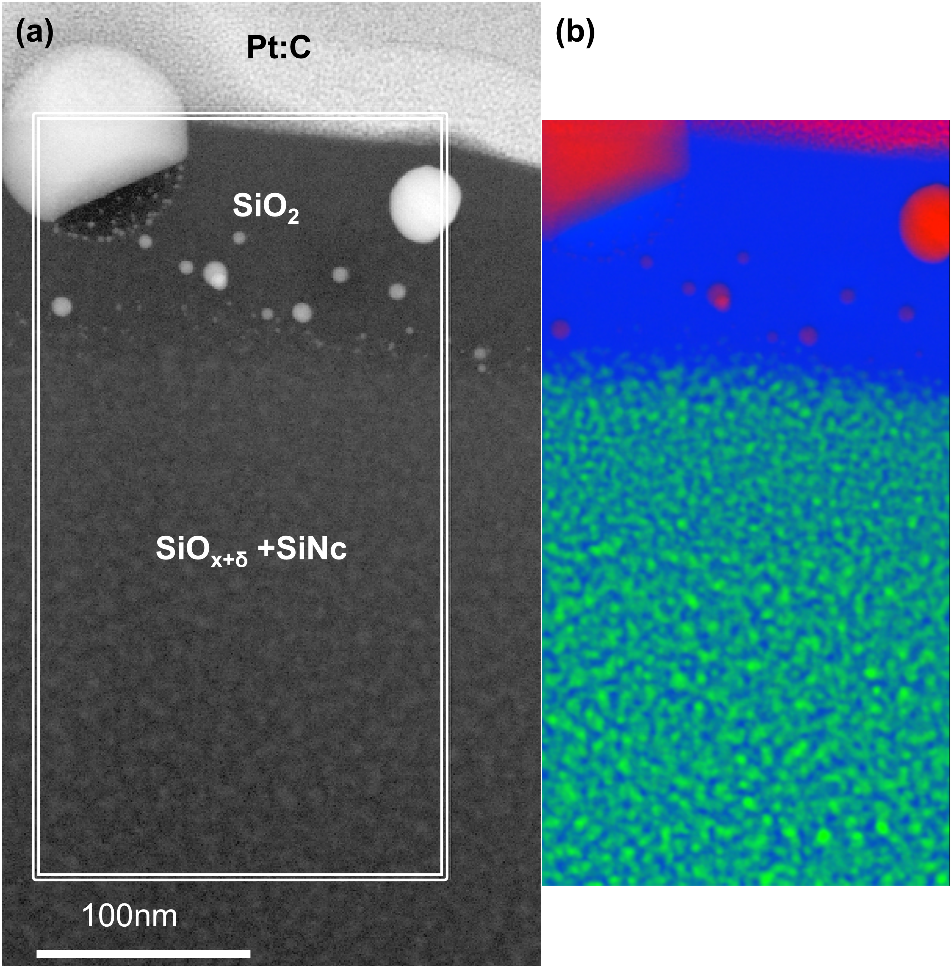


Figure SM3: AuNP distribution after annealing for 2 h in air and 1 h in N_2_ (A2_N1). (a) STEM-ADF AuNp of different size beneath the surface and at the interface between the oxidized SiO_x_ (labeled as ‘SiO_2_’) and the SiO_x_ decomposed into SiO_x+δ_ and SiNc. Please note, that compared to Figure SM2 AuNp close to the SiO_2_ – SiO_x+δ_ interface are smaller. (b) Color-coded contributions of Au (red), Si (green) and SiO2 (blue) to the EELS signal obtained by fitting a spectrum image.


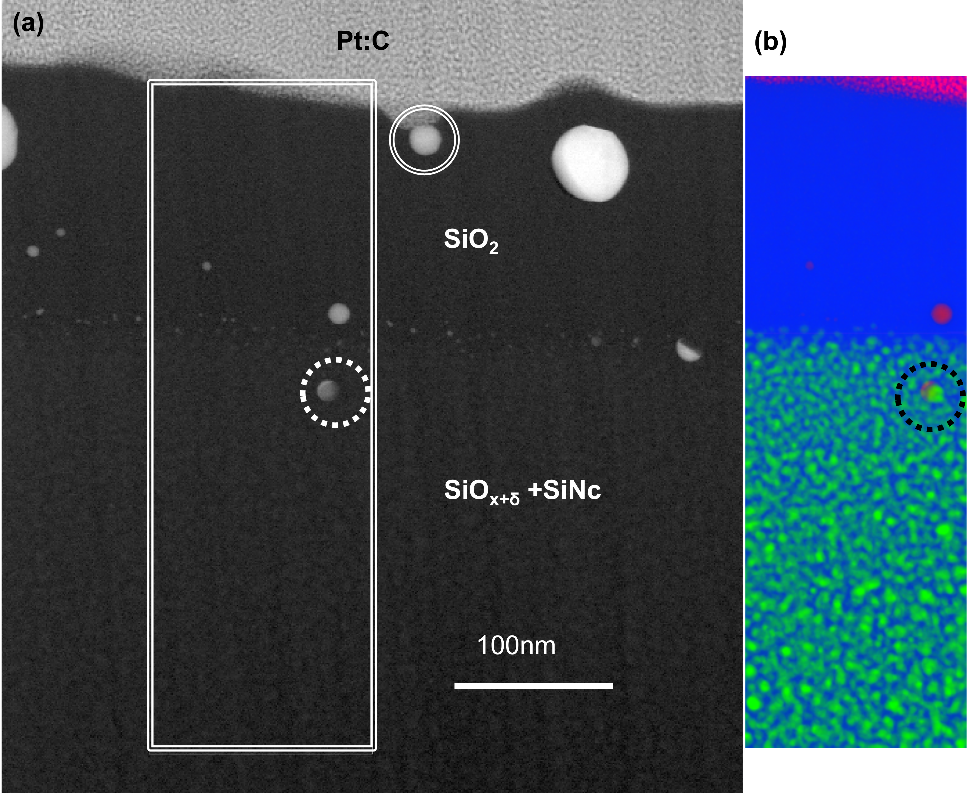


Figure SM4: AuNP distribution after annealing for 3 h in air (A3_N0). (a) STEM-ADF showing AuNp of different size beneath the surface and at the interface between the oxidized SiO_x_ (labeled as ‘SiO_2_’) and the SiO_x_ decomposed into SiO_2_ and SiNc. Please note, that compared to SM2 AuNp close to the interface SiO_2_ - SiO_x+δ_+SiNc are smaller and the overall density of AuNp has decreased. In addition, circles mark AuNp connected to a surface pore (full circle) or are located inside the SiO_x+δ_ layer (dotted circles); both will be referred to in the Sec. Discussion. (b) Color-coded contributions of Au (red), Si (green) and SiO_2_ (blue) to the EELS signal obtained by fitting a spectrum image.

*
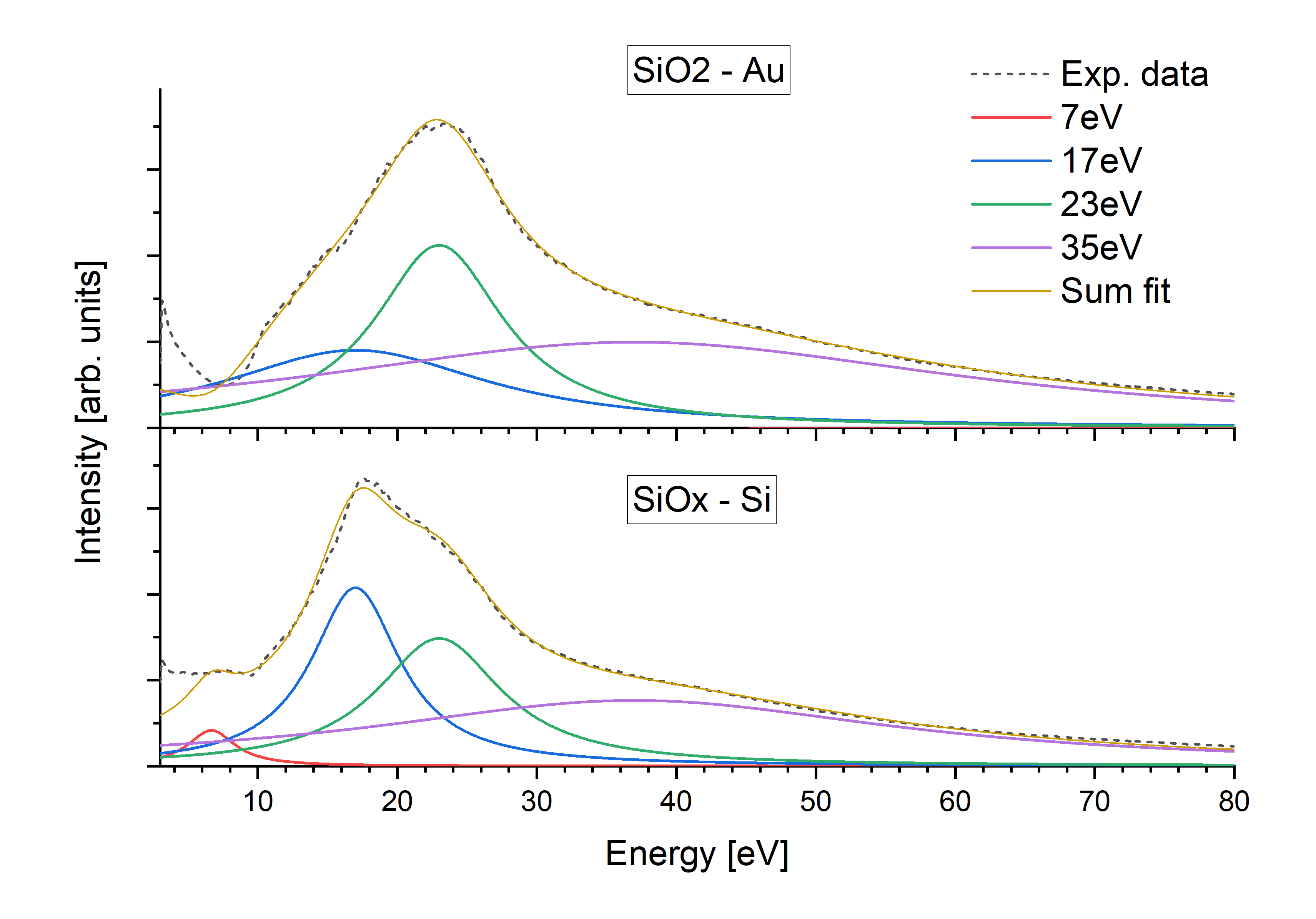
*

Figure SM5: Examples of the EELS spectral decomposition, both taken from average spectra of larger areas within the EELS maps. Top: mixed SiO_2_ and AuNp signal, bottom: mixed SiO_x_ and SiNc signal. Both spectra are decomposed via non-linear least-square fitting into multiple Lorentzian peaks with initial parameters centered around strong features. The weak feature at around 7 eV has been previously attributed to the surface plasmon of Si [K.A. Mkhoyan, T. Babinec, S.E. Maccagnano, E.J. Kirkland and J. Silcox, “Separation of bulk and surface-losses in low-loss EELS measurements in STEM” Ultramicroscopy, vol. 107, pp. 345-355, 2007, https://doi.org/10.1016/j.ultramic.2006.09.003]. This feature is absent in the SiO_2_ spectra, but is generally too low to be useful for fitting of the Si content in individual spectra and is only included to improve the fit of the subsequent volume plasmons. The peaks at 17 and 23 eV are assigned to Si-Si and Si-O, respectively [A. Eljarrat, L. López-Conesa, J. López-Vidrier, S. Hernández, B. Garrido, C. Magén, F. Peiró, and S. Estradé, “Retrieving the electronic properties of silicon nanocrystals embedded in a dielectric matrix by low-loss EELS,” Nanoscale, vol. 6, no. 1, pp. 14971-14983, 2014, https://doi.org/10.1039/C4NR03691C]. The relative height of each peak gives a qualitative measure for the concentration of pure Si to SiO_x_ in the spectrum, an exact quantitative treatment requires sophisticated modeling approaches [C. Spinella, C. Bongiorno, G. Nicotra, E. Rimini, A. Muscarà, and S. Coffa, “Quantitative determination of the clustered silicon concentration in substoichiometric silicon oxide layer,” Appl. Phys. Lett., vol. 87, issue 4, 2005, https://doi.org/10.1063/1.1999839]. The broad feature around 35 eV is related to a number of Au transitions in addition to a smoothly decaying background. Due to its width, it is best fitted by a Gaussian, but for the purpose of visualizing Au in the SiO_x_ matrix, the sum of the inelastic scattering and the incoherent high-angle scattering are correlated, so that the STEM-ADF signal can also be used.
